# Supplementary material for: Do cancer detection rates differ between transperineal and transrectal micro-ultrasound mpMRI-fusion-targeted prostate biopsies? A propensity score-matched study
Source: PLoS One. 2023 Jan 18;18(1):e0280262. doi: 10.1371/journal.pone.0280262 (PMC9847953; doi:10.1371/journal.pone.0280262)
Supplement: Attachment — Submitted filename: Response to reviewers.docx [file pone.0280262.s002.docx]

**Response to reviewers**

**Reviewer #1**

Reviewer #1: The authors retrospectively compared the overall prostate cancer (PCa) and clinically significant prostate cancer (csPCa) detection rate between transperineal (TP) and transrectal (TR) micro-ultrasound mpMRI-fusion-targeted prostate biopsies in a series of only 135 patients. The secondary outcomes were to compare the detection rates of random and targeted biopsies stratified per access route and to evaluate micro-US for its potential added value. They found that the PCa and csPCA detection rates between TR and TP were similar. Detection rates also did not differ between random and targeted biopsies stratified per access route TR vs. TP. Additionally; they reported that targeted micro-ultrasound biopsies upgraded 2% of patients to csPCa

The micro-ultrasound is a promisor new technique that may improve PCa diagnosis and therefore new data is welcome. The novelty of this study relies in the comparison of TP vs. TR route employing MRI and micro-ultrasound. The sample size is small and this is the main problem of this study - 47 vs. 88 cases make hard to anyone to conclude that both biopsy routes are similar. The authors recognize this issue and justify by saying that this paper represents their initial results. Based on that I recommend rejection until a larger sample is achievable by this group. The paper has also some methodology problems that require major revision if the editor consider acceptance of it.

Major revisions

1) If the main purpose is to compare the detection rate of TR versus TP biopsy; the exclusion of anterior zone tumors definitively compromise the results and probably is the main cause of no detection rate difference between the two groups. If I agree with the removal of anterior tumors at MRI because it would favor the TP group (author´s justification), I also should remove posterior tumors in accordance because it would favor the TR route.

Thank you for this question. While we appreciate the provocative suggestion of the reviewer, we would like to clarify this issue. In this study, we could not compare the cancer detection rate of targeted TR-B vs TP-B for lesions located in the anterior part of the prostate because in our center this was per se an indication to TP biopsy. This decision was based on the evidence, that clearly favors TP route for anterior cancer detection. This is noted in the methods and in the discussion section of the paper as follows:

*“In our study, we did not compare the anterior lesion cancer detection rate between the two approaches as the presence of an anterior lesion was per se an indication to TP-B.”*

*“Second, our patients with anterior lesions systematically underwent TP-B; therefore, we could not compare the clinically significant disease detection rates in anterior zones between the two approaches.”*

Also, with regard to the reviewer’s point, there is no evidence to our knowledge that the TR route would favor posterior cancer detection. On the contrary studies directly comparing the two approaches do not show any significant difference between TR and TP for posterior cancer detection [1].

2) The small sample size is the major limitation of this study. They reported on only 135 patients of which ~42-45% of cases were positive for csPCa. This small number of cases probably jeopardize the study’s results and the conclusions. For example, the csPCa detection rate between random and MRI guided were similar in this trial, which is against the whole body of the MRI guide prostate biopsy literature that shows that MRI guided fragments have a higher chance of csPCa when compared to random fragments. The small number of cases may explain this contradictory finding

While we have acknowledged in the manuscript that the small sample size is a limitation of the present study, our results are completely in line with the literature showing that systematic biopsy detects more insignificant disease, but less significant disease as compared to MR-targeted biopsy. This difference is statistically significant both in the TR and in the TP subgroups.

In the transrectal biopsy subgroup, GG1 disease was detected in 54% of all positive cases by systematic biopsy vs 24% of all positive cases by targeted biopsy. GG2≥ disease was detected in 46% of all positive cases by systematic biopsy vs 76% of all positive cases diagnosed by MR-targeted biopsy (p = 0.005).

The same was observed in the transperineal biopsy subgroup. GG1 disease was detected in 46% of all positive cases by systematic biopsy vs 27% of all positive cases by targeted biopsy. GG2≥ disease was detected in 54% of all positive cases by systematic biopsy vs 73% of all positive cases diagnosed by targeted biopsy (p = 0.03).

To further clarify this important issue, we have added to the following phrases to the results section:

*“Targeted biopsies detected more clinically significant disease and less clinically insignificant disease than systematic biopsy in both approaches (TR-B p < 0.01; TP-B p = 0.03) on a patient level.”*

3) The author say that this is their preliminary result but the last case was included two years ago. Once the sample is of only 135 cases, why the authors did not include the most recent performed cases?

Thank you for this comment. Due to the risk of infections complications we completely abandoned the transrectal approach in 2020 [2]; therefore, we were unable to further increase the sample size.

4) The add value of micro-ultrasound was of only 2% but even so the authors came to the conclusion that it has an additional diagnostic value over systematic and MRI-targeted biopsies. I don´t think this 2% is clinically significant. For example, it is acknowledge that a biopsy can be avoided in patients with PIRADS-2, previous negative biopsy and a PSA density below 0.15 because in this situation we would miss only 4 to 5% of csPCa. If missing 5% of csPCa is acceptable I don´t think that an increase of only 2% by adding micro-ultrasound has any clinical benefits. According to the results, I would have to employ micro-ultrasound in 50 cases to diagnose an additional csPCa case. Once these are the initial results perhaps, the added micro-ultrasound value may be higher with the inclusion of more cases.

8) The micro-ultrasound detected 36 MRI invisible lesions in 33 patients but only 19% of them were CsPCa at biopsy result and the final additional benefit in increasing csPCa detection was of only 2%. Where is the micro-ultrasound advantage? 67% of suspicious lesions at micro-ultrasound had no cancer at all......

Thank you for these comments which all relevant and pertinent to the discussion. While we think we have provided our views on this subject in the discussion section, we are happy to further explore this argument. Although we completely agree with the reviewer that the added diagnostic value of 2% seems to be trivial, we would argue that the high overall cancer detection rate in our study is partly a result of the value of micro-US, which enables an accurate definition of prostatic anatomy and might reduce cognitive, or software errors observed with other MRI fusion strategies. From our subjective experience, the available mpMRI-micro-US fusion system (FusionVu^TM^) helps to identify the region of interest in the prostate. The visibility of most lesions in the peripheral areas on microUS allows an intraoperative quality control which cannot be achieved with registration strategies. Obviously, the findings of the present study need to be validated in prospective trials; hopefully, the OPTIMUM trial will clarify the added value of microUS in a rigorous manner[4].

5) At methodology line 103, the author say that all MRI was reviewed which is important. However, it is not clear how this was done? How many radiologists participated at this “uroradiology meeting”? The uroradiologists had experience reading multiparametric MRI? If there is disagreement regarding the assigned PIRADS how this was solved?

Thank you for this helpful comment. In our center each MRI is read and scored by a single expert radiologist with more than 15 years of experience in prostate MRI. We added the following phrase to the Methods section:

*Prior to biopsy, all mpMRI were reviewed in a dedicated uroradiology meeting where a single expert radiologist with over 15 years’ experience in prostate MRI (JYM) annotated the relevant lesions.*

6) Despite the propensity score-matched process the groups are still unbalanced. The transperineal group has a higher number of sampled fragments. The author think that this may have an impact at detection rate between the two groups?

Thank you for your question. TP approach was used in some patients to determine patient eligibility for focal therapy. In these patients, additional systematic biopsies were taken in the anterior part of the prostate to exclude MR nonvisible significant lesions. This is probably the main reason why the mean total TP biopsy number was higher than total TR biopsy number. We would also like to highlight that the median targeted biopsy number was the same between the two approaches.

Also, the propensity matching was not performed for the number of sampled fragments. We selected the baseline patients’ characteristics prior to the biopsy (age, PSA, clinical stage, prostate volume, mpMRI score (PI-RADS 1-2 vs 3-5), number of mpMRI-visible lesions and indication to biopsy).

Finally, we do not think that the difference in total biopsy cores between the two approaches had a significant impact on the results for detection of significant disease. The additional cores were always taken from the MRI and micro-US negative areas of the prostate.

7) At discussion and conclusion the author highlighted the potential benefits and accuracy of micro-ultrasound that are not based in their results. They argue that TR and TB detection rates were similar because the benefits of micro-ultrasound. However, both groups underwent biopsy with micro-ultrasound therefore what is been compared is not the advantage of micro-ultrasound but the difference of TR versus TP route. A third transrectal and a fourth transperineal arms without micro-ultrasound would be necessary to attest that the lack of difference between groups 1 and 2 were due the advantage of micro-ultrasound or its higher accuracy.

Thank you for your comment. The reviewer is correct that a study design with additional third and fourth transrectal and transperineal arms would be necessary to confirm our statement. Unfortunately, this was not the intended study design or a technical possibility in our department.

However, our hypothesis is based on the high and similar overall cancer detection rate in both arms. In other words, when using micro-US, the route of biopsy is not as important as the technology itself. Ideally, our findings should be confirmed in a multi-arm and randomized prospective study.

Minor revision

1) Why the table 1 that compare the patient´s characteristics between the two groups do not contain the p-values? The p-values must be included at tables and not only at the text.

Thank you for your comment. We added the p values to the patients’ characteristics table (table 1) We would also like to highlight that we did include the phrase that standardized mean differences were all below < 0.1 in the results section [5,6].

2) I think table 3 is unnecessary

While we respect the reviewer’s point of view, we believe that this table improves the completeness of the manuscript. We would agree to revise if the editorial board wishes so.

3) This study is a hypothesis generating study and this should be highlighted at study limitations at discussion section (lines 260 -267).

Thank you for your comment. We highlighted this in the limitations section as suggested.

*“First, it’s a retrospective and a hypothesis generating study that is prone to selection bias.”*

4) Line 119 change “TR-P” to “TP-B”

Thank you for your remark. We made the requested change.

5) MRI was “positive” in 60% of cases (line 168) How many cases were micro-ultrasound positive I mean PRIMUS 3 to 5?

Thank you for your question. In this study, we did not assess the correlation of concordant significant disease between the micro-US and MRI visible lesions. However, our initial results with lesions by lesion analysis are available in another publication [3]. In all patients, 73% of mpMRI lesions were visible with micro-US.

6) Why to mention the Future trial that did not compared TP versus TR biopsies ? Instead they compared Fusion vs. Cognitive vs. in Bore biopsy? The Future did not evaluate micro-ultrasound either. Furthermore it is well known that Future trial is an underpowered study because at sample size calculation of the trial they needed around 400 PIRADS 3-5 lesions but they had only 234 cases with suspicious MRI.

Thank you for your comment. We agree with the reviewer and we removed the FUTURE trial reference.

**Reviewer #2:** It is an interesting and present issue. The microultrasound have been shown promissed results and probably will have a role in prostate cancer diagnostic scenario. The authors report results from a retrospective matched groups comparison which showed no difference between detections rates between transperineal and transrectal prostate biopsies using micro-ultrassound. Despite the retrospective design study decreases its conclusions streght these results deserve to be published. The choice for a prostate biopsy route should take the detection rate in consideration. This study reinforce a tendency showed in previous publications which compared perineal and transrectal route using ordinary ultrasound devices.

Thank you for your kind comments.

1) Do the authors believe that the report of anterior-only positive biopsies on perineal route could be interesting for comparison between perineal and transrectal detection rates?

Thank you for your question. We think that an anterior only lesion comparison would clearly favor the transperineal approach based on the current literature and our experience. This was the main reason why an anterior MRI lesion is a per se indication to a transperineal biopsy in center. Please see also R1 to reviewer 1 for additional comments on this subject.

2) Regarding the low PPV value of PIRADS 3 MRI targeted biopsies, do the authors believe that a secondary analysis including only PIRADS 3 splited from PIRADS 4-5 could improve the results? How many PIRADS 3 lesions presented different characteristics on micro-US views, for example? Thinking in this issue, In Table 1 - Patients Characteristics, could the rate of PIRADS 3 be individualized?

Thank you for your question. Only a minority of patients had PIRADS 3 positive lesions (5 patients out of 81 or 6% of all patients with suspicious MRI lesions). We did not assess individual MRI and micro-US lesion correlation in this study, but our initial experience on this question is published in a separate paper [3]. While we believe that this comment is really pertinent, we do not think that excluding these 5 patients would have any significant difference in our results.

3) Were the micro-US target biopsies performed by a blinded operator? Specifically, had the radiologists previous access to the MRI images, before biopsy? Do the authors believe that this fact could interfer in positive rates of micro-US target biopsies when compared to MRI fusion biopsies?

Thank you for your question. No, the operator was not blinded to the MRI results and yes, the radiologist had the access to the MRI images prior to the biopsy and marked the suspicious lesions. We would like to highlight the following phrase from the methods section.

*“Prior to biopsy, all mpMRI were reviewed in a dedicated uroradiology meeting where a single expert radiologist with over 15 years’ experience in prostate MRI (JYM) annotated the relevant lesions.”*

We fully agree that prior knowledge of the MRI results before the biopsy generates a procedure bias when comparing the diagnostic performance of micro-US-MRI fusion biopsy vs micro-US biopsy alone. This question is currently being assessed in the OPTIMUM trial [4]. However, this was not the aim of our study. Our goal was to assess if the micro-US have any additional value in a setting where the surgeon is not blinded to the MRI rapport and compare the results between the TP and TR biopsy approaches

4) Do the rates of micro-US targeted biopsies differ from random biopsies? In my opinion, it is not clear in the report.

Thank you for your question. The micro-US visible and MRI nonvisible lesion targeted biopsies were performed in 33 patients (24%). In total, 11 patients had cancer detected based on micro-US only. Out of these patients clinically non-significant disease was seen in 5 (45%), and clinically significant disease was seen in 6 (55%). These results are similar to the random biopsies of our series; however, the sample size is quite small, and we do not think we can draw any conclusions.

[1]	Pepe P, Garufi A, Priolo G, Pennisi M. Transperineal Versus Transrectal MRI/TRUS Fusion Targeted Biopsy: Detection Rate of Clinically Significant Prostate Cancer. Clin Genitourin Cancer 2017;15:e33–6. https://doi.org/10.1016/j.clgc.2016.07.007.

[2]	Loeb S, Vellekoop A, Ahmed HU, Catto J, Emberton M, Nam R, et al. Systematic review of complications of prostate biopsy. Eur Urol 2013;64:876–92. https://doi.org/10.1016/j.eururo.2013.05.049.

[3]	The benefit of adopting Microultrasound in the prostate cancer imaging pathway: A lesion-by-lesion analysis: Biopsies prostatiques guidée par micro-échographie, quel bénéfice ? Une analyse lésion par lésion - ScienceDirect n.d. https://www.sciencedirect.com/science/article/pii/S1166708722001725?via%3Dihub (accessed September 24, 2022).

[4]	Klotz L, Andriole G, Cash H, Cooperberg M, Crawford ED, Emberton M, et al. Optimization of prostate biopsy - Micro-Ultrasound versus MRI (OPTIMUM): A 3-arm randomized controlled trial evaluating the role of 29 MHz micro-ultrasound in guiding prostate biopsy in men with clinical suspicion of prostate cancer. Contemp Clin Trials 2021:106618. https://doi.org/10.1016/j.cct.2021.106618.

[5]	Austin PC. Optimal caliper widths for propensity-score matching when estimating differences in means and differences in proportions in observational studies. Pharm Stat 2011;10:150–61. https://doi.org/10.1002/pst.433.

[6]	Austin PC. The use of propensity score methods with survival or time-to-event outcomes: reporting measures of effect similar to those used in randomized experiments. Stat Med 2014;33:1242–58. https://doi.org/10.1002/sim.5984.
